# Experimental Evaluation of Different Optical Fibers for Micro-Invasive Soft-Tissue Ablation with a 1064 nm Diode Laser System

**DOI:** 10.3390/s26031073

**Published:** 2026-02-06

**Authors:** Danny Di Minno, Cosimo Trono, Lorenzo Capineri, Alessia Blundo, Giovanni Masotti

**Affiliations:** 1 Department of Information Engineering, University of Florence, 50139 Florence, Italy; danny.diminno@edu.unifi.it; 2 Institute of Applied Physics “Nello Carrara”, National Research Council (CNR_IFAC), 50019 Sesto Fiorentino, Italy; c.trono@ifac.cnr.it; 3 Elesta S.p.A., 50041 Calenzano, Italy; a.blundo@elesta-echolaser.com (A.B.); g.masotti@elesta-echolaser.com (G.M.)

**Keywords:** balloon catheter, diode laser, laser ablation, optical fibers, prostate treatment, soft tissue

## Abstract

**Highlights:**

**What are the main findings?**

- Comparison of different optical fibers for soft-tissue laser ablation.
- Light diffusion by a balloon catheter with hydroxyapatite powder.

**What are the implications of the main findings?**

- A quantitative reference framework for selecting and designing optical fibers.
- Circumferential ablation in confined anatomical spaces.

**Abstract:**

This study presents an experimental evaluation of different optical fibers for soft-tissue laser ablation using an Echolaser system, developed by Elesta S.p.A., for minimally invasive therapies. Eight fibers with varying core diameters, numerical apertures, and tip geometries (flat, conical radial, and spherical) were compared to investigate the influence of optical properties on the ablation dimensions and thermal profiles. The experiments were conducted at 1064 nm with powers of 3, 5, and 7 W and delivered energies ranging from 1200 to 3600 J. The results highlight how the fiber characteristics affect tissue ablation, identifying the configurations suitable for minimally invasive prostate applications. These findings provide an experimental reference for the development of laser-based biomedical approaches.

## 1. Introduction

Over the past decades, laser systems have found increasing applications in biomedical fields, offering controlled, selective, and minimally invasive energy delivery for the treatment of various pathologies [1]. Optical fibers play a key role in these applications by guiding laser radiation to target tissues in a controlled manner.

In urology, minimally invasive thermal ablation techniques are increasingly being explored for the treatment of pathologies, such as benign prostatic hyperplasia (BPH) and localized prostate cancer. BPH, in particular, affects a significant proportion of aging men, and its management often requires tissue debulking to relieve urinary symptoms. Traditional surgical options, though effective, can be associated with bleeding, prolonged recovery, and functional side effects. In contrast, focal laser ablation represents a promising alternative, enabling precise delivery of energy through optical fibers to induce coagulative necrosis with limited harm to the surrounding structures [2]. This minimally invasive approach allows for treatments to be tailored to a patient’s pathology, ranging from debulking to complete ablation, while preserving the surrounding healthy tissue and critical anatomical structures. Furthermore, its versatility, reproducibility, and compatibility with advanced planning and guidance systems make it a safe and efficient option for a variety of clinical scenarios [3].

Various modalities beyond laser ablation have been applied clinically, including radiofrequency ablation (RFA) [4], microwave ablation (MWA) [5], high-intensity focused ultrasound (HIFU) [6], and cryotherapy [7], which can be used for focal treatments of the prostate. Among these energy-based techniques, microwave ablation (MWA) has attracted increasing interest in recent years. While microwave systems can provide rapid heating and relatively large ablation volumes, they are often associated with increased system complexity and higher costs. Moreover, the reduced sensitivity of microwave energy deposition to a tissue’s electrical properties, although advantageous in some settings, may limit fine control of the spatial energy distribution, potentially resulting in less precise ablation margins. High intratumoral temperatures and large ablation zones may also increase the risk of collateral thermal damage, particularly in anatomically confined regions or near critical structures. These limitations highlight the need for alternative minimally invasive techniques, such as focal laser ablation, which offer improved spatial selectivity and controlled energy delivery [3].

The monitoring of tissue temperature during laser ablation treatment is fundamental for the correlation of tissue property changes and the evaluation of the volume affected by the laser ablation. Non-contact real-time temperature measurements are less invasive, and infrared imaging [8,9] and MRI thermometry [10,11,12,13] are the two most promising techniques. Infrared imaging is an affordable method but needs calibration for the different emissivities of surface tissues and provides only a 2D projection of the 3D heated volume; an MRI is a real-time 3D technique but is more expensive and less appreciated by some patients suffering from claustrophobia. Contact methods, like the point-like temperature probes (thermocouples [8,14] or thermistors) of fiber-optic temperature sensors [9], are more accurate but more invasive. Often, two methods can be combined in a laboratory; for example, using a thermocouple for calibrating the infrared camera on a point reduces the uncertainty regarding the surface emissivity. The temperature field mapping of a solution with five Fiber Bragg Grating temperature sensor arrays is proposed in [9]: this solution can achieve high resolution at 0.1 °C and samples the temperature in a reasonably dense 3D matrix of positions to investigate the uniformity of the treated volume, especially when the laser tip is close to a vessel. All these techniques are reported to provide the reader with feasible solutions for further laboratory investigations.

Moreover, optical fibers have already successfully demonstrated their low invasiveness in various applications involving the analysis of biological tissues [15].

However, each of these techniques still presents with important limitations. For instance, HIFU, despite its non-invasiveness, has shown variable oncologic control and functional outcomes, with its long-term efficacy remaining debated [6]. Cryotherapy, which relies on repeated freeze–thaw cycles, may result in collateral tissue injury and requires careful probe placement to minimize damage to adjacent structures [7]. More broadly, systematic reviews have indicated that focal therapies, including laser-based approaches, are generally safe and effective for preserving function; however, oncologic outcomes and recurrence rates remain highly variable across techniques, patient selection criteria, and study designs [4].

In laser-based approaches, most clinical implementations use conventional flat-tip fibers, which emit light in a forward direction and often lead to elongated ablation zones rather than more uniformly shaped lesions, as observed in our experiments. Alternative fiber geometries, such as conical or spherical (ball) tips, have been proposed to improve the energy distribution; however, systematic experimental evaluations in biological tissues are still limited in the present study.

Moreover, important fiber parameters, like the core diameter and numerical aperture, have not been exhaustively studied in combination with the tip shape to understand their impact on ablation morphology and thermal behavior. This knowledge gap hinders the design of optimized fiber-based delivery systems for more predictable and efficient ablation.

In this study, we present a systematic experimental comparison of eight different optical fibers varying in core size, numerical aperture, and tip geometry (flat, conical radial-emitting, and spherical). Using a calf liver as a tissue surrogate for a human prostate due to its similar optical and thermal properties, we perform ablation experiments with a diode laser system (1064 nm) integrated into the Echolaser platform [16], evaluating single-fiber, dual-fiber, and pull-back irradiation strategies. We further explore a novel method to achieve uniform, circular ablations by coupling a flat-tip fiber with a balloon catheter filled with a hydroxyapatite-based diffusing medium.

Our results provide new insights into how fiber design influences ablation volume and temperature distribution, with potential impact for the development of minimally invasive, fiber-based laser therapies with urological applications.

## 2. Materials

### 2.1. Instruments Used and Optical Fibers Tested

All the experiments were conducted at the Elesta Laboratory in Calenzano (Italy) using the following instrumentation:

1. Two Echolaser X4 systems were employed: one unit was used for the tests involving Elesta–Oberon (Calenzano, Italy) optical fibers equipped with an SMA905 standard connector featuring a 0.1 mm setback, while the second unit was used for the tests performed with Oberon (Wildau, Germany), Thorlabs (Newton, NJ, USA) or Asclepion (Jena, Germany) optical fibers with a standard SMA905 connector;
2. Ultrasound imaging was carried out using an Esaote MyLabOmega system equipped with a linear probe;
3. Temperature monitoring was performed using an Agilent Data Acquisition/Switch Unit (SN US37034947) (Keysight Technologies, Santa Rosa, CA, USA) in combination with two type-K thermocouples: one RS thermocouple (SN 6212170) (RS Components GmbH, Bad Hersfeld, Germany) with a 0.3 mm probe diameter, 2 m length, and +600 °C range mounted on a 14 G needle, and a second RS thermocouple (SN 8047990) (RS Components GmbH, Bad Hersfeld, Germany) with a 0.076 mm probe diameter, 2 m length, and +260 °C range mounted on a 21 G needle;
4. A Tsunami Medical (Mirandola, Italy) balloon catheter (PBKK 11/20-20; outer diameter 11 G, length 20 cm, balloon volume 5.5 mL) and a Tsunami Medical inflation kit (RK07) were used to perform the balloon-assisted procedures;
5. The optical power measurements were obtained using an Ophir (Jerusalem, Israel) power meter (7Z01565);
6. The optical fibers were prepared using a Jonard Tools (Elmsford, NY, USA) adjustable wire stripper (20–30 AWG, ST-500).

Eight multimode optical fibers were evaluated. Their characteristics are summarized in Table 1.

Flat tip (Fiber ID: N°1, N°2, N°3, N°4 and N°5):

The flat-tip fibers exhibited a single central lobe in the polar diagrams, indicative of a predominantly axial emission with an approximately Gaussian-like angular distribution.

In these fiber types, increasing the numerical aperture (NA) from 0.22 to 0.48 resulted in a progressive enlargement of the divergence angle, leading to a broader irradiated spot and consequently a reduction in the power density.

Variations in the core diameter (from 272 µm to 600 µm) mainly affected the initial spot size, while the overall lobe shape remained essentially unchanged.

Conical tip (Fiber ID: N°6 and N°7):

The conical-tip fibers with radial emission exhibited two opposite lateral lobes with a minimal axial component. This behavior is consistent with the conical geometry, which refracts the guided radiation toward the lateral directions.

Curved ball tip (Fiber ID: N°8):

The curved ball-tip fiber features a spherical distal termination with a curvature toward one side.

The corresponding polar diagram in Table 2 shows a right-shifted emission, resulting from beam deviation induced by the curvature of the spherical tip. The emitted radiation is diffuse but not isotropic, exhibiting a pronounced directional component. Such a characteristic may be advantageous in applications requiring selective illumination of specific regions within an optical field.

### 2.2. Thermocouples for Temperature Measurements

The Temperature was measured using a type-K thermocouple placed inside a 14 G, 18 G or 21 G needle positioned:

1. 10 mm from the fiber tip in the single-fiber tests;
2. At the midpoint between the fibers in dual-fiber configurations.

**Figure 1:**
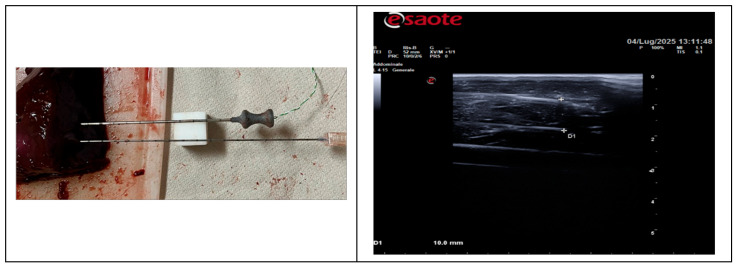
(**Left**) Teflon block for keeping the needles with optical fibers and the microthermocouple at a distance of 10 mm. (**Right**) Echographic image for monitoring the actual distance of the two tips of the needles indicated by the crosshairs; cursor D1 indicates distance equal 10 mm.

During the experimental tests, a type-K microthermocouple was inserted into the tissue (pork fillet or calf liver) using an introducer needle, positioned and maintained parallel to the optical fiber by a TEFLON block, which was also inserted using a separate needle, at a mutual distance of 10 mm (see Left, Figure 1). The correct positioning of the optical fiber and the thermocouple was performed under ultrasound guidance, using a linear ultrasonic probe applied in contact with the tissue and interposing ultrasound gel as an acoustic coupling medium (see Right image in Figure 1).

### 2.3. Laser System

All the tests were performed using an Echolaser (Elesta S.p.A.), a 1064 nm diode laser system. The system integrates a laser source, a guiding ultrasound interface, and dedicated control software (Echolaser Smart Interface, ESI), allowing for precise planning, real-time monitoring, and verification of treatment parameters [16]. The operating parameters for this study included output powers of 3 W, 5 W, and 7 W, with delivered energies ranging from 1200 to 3600 J.

### 2.4. Angular Optical Power Distribution Measurement System (Goniophotometer)

The angular emission profiles were measured using a goniophotometer system composed of:

1. A dark chamber;
2. A rotation stage controlled by a stepper motor;
3. A photodiode sensor mounted on a rotating arm.

The acquisition of the polar emission diagrams for each fiber was performed by first recording the initial reference position of the motorized stage supporting the photosensitive detector connected to the goniophotometer’s control software. The procedure began with a measurement of the background noise level in a dark room. Subsequently, for each fiber, an optical power of 1 W was delivered for the time required to sample the 400 angular points predefined by the software, with an angular spacing of 0.5° between consecutive measurements.

## 3. Methods

### 3.1. Optical Fiber Emission Theory—Numerical Aperture (NA)

The emission characteristics of an optical fiber are mainly determined by its core diameter, numerical aperture (*NA*) and by the geometry of the distal tip. The numerical aperture defines the maximum acceptance (and emission) angle of the fiber and is expressed as

(1) $$NA=n_{0\;}sin\left(\theta_{max}\right)=\sqrt{{n_{1}}^{2}-{n_{2}}^{2}}$$

where $n_{1}$ and $n_{2}$ are the refractive indices of the core and cladding, respectively, and $n_{0}$ is the refractive index of the external medium.

The *NA* therefore determines the divergence of the output beam: fibers with a higher *NA* produce a larger emission cone, whereas fibers with a lower *NA* generate a more collimated beam.

### 3.2. Laser Ablation Phenomenon, Radiation–Matter Interaction and Thermal Effects

Laser–tissue interaction produces localized heating, leading to coagulative necrosis, which starts around 60 °C and progresses with the exposure time [17]. The resulting ablation volume in the case of a flat-tip fiber is initially ellipsoidal and diverges radially until reaching a saturation plateau. The energy delivery, number of fibers, and spacing determine the final ablation size. The primary laser source is a diode emitting at λ = 1064 nm, with a circular beam profile, maximum output of 7 W per fiber, numerical aperture *NA* = 0.22, and continuous-wave emission. This wavelength provides optimal penetration of soft tissues while maintaining sharp ablation boundaries, due to the lower absorption coefficient compared to alternative wavelengths (e.g., 532 nm, 980 nm, 1480 nm). A visible red diode (635–670 nm) is also included for aiming purposes.

### 3.3. Sample Selection and Fiber and Thermocouples Insertion Techniques

Two tissue models were used:

1. Calf liver: commonly used substitute for prostate tissue due to comparable optical/thermal properties;
2. Porcine muscle for preliminary verification tests.

The values reported in Table 3 show that the thermal and optical properties of the three tissues are considered to fall within the typical ranges reported in the literature for biological tissues; a set of references is reported in [18,19,20,21,22,23,24,25,26,27,28,29,30,31]. In particular, the density, specific heat, and thermal conductivity of the prostate, liver, and porcine muscle are similar, while the water content shows more marked differences: the prostate and liver present higher values than the porcine muscle, the latter being a lean and more fibrous muscle. This makes the liver and prostate more comparable, especially regarding their thermal and optical responses to a laser.

The diffusion coefficient reported in Table 3 refers to the effective spread of light within the tissues at λ = 1064 nm. It accounts for both the absorption and scattering properties of the tissues, indicating how far light penetrates before being absorbed.

Regarding the optical properties at 1064 nm, it can be observed that the prostate and liver present with comparable absorption and scattering values, while the porcine muscle stands out for significantly lower values, particularly in the diffusion coefficient. An ex vivo calf liver, which shares some optical and thermal similarities with the prostate, was chosen as an ex vivo model for laser ablation because its properties at near-infrared wavelengths are well characterized, and its homogeneous structure and high-water content allow for the reproducible investigation of laser-induced thermal lesion formation.

Overall, the analysis confirms that the liver, thanks to its greater similarity to the prostate in terms of water content, composition and optical–thermal properties, represents a more suitable model for studying laser ablation phenomena.

The optical fibers and thermocouples were inserted into the tissue using needles, whose dimensions were matched to the outer diameter of the fibers and of the thermocouples. Specifically, 21 G, 18 G, and 14 G needles were used for the fibers, while 21 G and 14 G needles were employed for the thermocouples. The distance between the fibers and thermocouples was set at 10 mm, based on preliminary experiments that indicated this distance as appropriate for positioning the sensors at the periphery of the expected maximum ablation zone.

### 3.4. Recognition of Ablation Size

**Figure 2:**
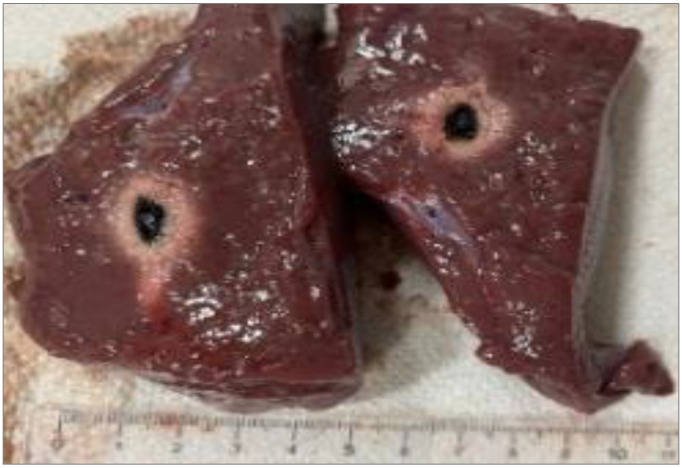
Single Fiber N°1 (5W@1800J). L = [19.3 ± 1.0] mm and W = [12.5 ± 0.6] mm (5W@1800J, 4 samples tested).

After sectioning the ablated tissue with a scalpel, an image of the sample was acquired with a ruler placed alongside it to record the scale for dimensional quantification, as shown in Figure 2. The acquired image was then processed on a computer, where an ellipsoid was overlaid onto the ablated region. Using the adjacent ruler, the dimensions L and W (representing the longitudinal and transverse axes of the ellipsoid) were subsequently measured.

The ablation zone is not defined as the carbonized core alone (blackened region), but as the entire tissue volume affected by laser-induced thermal damage. This includes both the central carbonized region and the surrounding heated tissue, which is consistent with a peripheral zone of coagulative necrosis.

The boundaries of the ablation zone were identified based on a macroscopic visual contrast, allowing for a clear distinction between the thermally damaged and unaffected tissues. Consequently, the ellipsoidal overlays and the reported ablation dimensions refer to the full extent of thermally induced tissue damage rather than only the visibly carbonized core.

### 3.5. Tissue Irradiation Techniques

In our study, the following experimental configurations were examined:

1. Single fiber;
2. Dual fiber with 10 mm spacing;
3. Pull-back technique (fiber retraction after initial energy delivery);
4. Balloon catheter with scattering medium (hydroxyapatite suspension) in selected trials.

Depending on the treatment protocol, up to four fibers can be used simultaneously to achieve the controlled ablation volumes, with real-time ultrasound guidance and ESI software enabling visualization of needle trajectories, fiber and thermocouples placement, and ablation zones before and during irradiation. The system supports different treatment strategies, including:

1. Single-fiber and multi-fiber configurations, to adapt the ablation volume and shape to the tissue geometry;
2. Pull-back technique, in which the fibers are retracted after initial energy delivery to extend the ablation region longitudinally.

The laser powers applied via the optical fibers were set at 3 W, 5 W, and 7 W, corresponding to delivered energy doses ranging from 1200 J to 3600 J, with 1800 J being the most commonly used value. The irradiation time and, consequently, the duration of each experiment varied depending on the chosen power and energy dose. For a dose of 1800 J, the test duration was 10 min at 3 W, 6 min at 5 W, and 4 min and 17 s at 7 W. These parameters were selected to deliver controlled energy to the tissue while ensuring reproducible ablation volumes, and their effects are further analyzed in the Results Section.

### 3.6. Outcome Parameters

The primary outcome measures are:

1. The longitudinal ablation size (L), as shown in Figure 2;
2. The transverse ablation size (W), as shown in Figure 2;
3. The temperature profiles within the tissues during laser ablation;
4. The radiation profile of the balloon catheter with Fiber N°1 inside and containing a solution made of hydroxyapatite and saline solution.

## 4. Results

### 4.1. Radiation Profiles of the Eight Types of Optical Fibers Analyzed

A result of this investigation is the comparison of radiation profiles. Table 2 reports the radiation profile of the eight optical fibers used in the experimental phase, as described in the previous sections.

Table 2 displays the polar diagrams representing the radiation profiles emitted by the eight different optical fibers used in this study. The physical quantities represented in the plots are the angular position and the emitted optical power. The diagrams were recorded using a goniophotometer with a scanning step of 0.5° and a laser power set to 1 W, allowing for the measurement of the angular distribution of light intensity for each fiber.

### 4.2. Optical Fibers with Flat-Tip Geometry

The ablation volumes increased with both the core diameter and numerical aperture (NA).

An increase in the core diameter results in an increase in both the longitudinal dimension *L* and the transverse dimension *W*, with a more marked impact on the dimension *L*. Regarding the numerical aperture, a higher aperture leads to a greater divergence of the output beam, translating into a prevalent increase in the transverse dimension *W*.

1. Core = 272 µm (Fiber N°1)—300 µm (Fiber N°2), *NA* = 0.22: Produced the smallest lesions, with the longitudinal dimensions (L) typically exceeding the transverse width (W), yielding an elongated, ellipsoidal profile.
2. Core = 300 µm (Fiber N°3)—400 µm (Fiber N°4), *NA* = 0.37: Resulted in a marked increase in W and a more spherical ablation shape. The transition from NA 0.22 to 0.37 produced the largest relative increase in W among all the pairwise comparisons.
3. Core = 600 µm (Fiber N°5), *NA* = 0.48: Generated the largest ablations overall. At 7 W, these fibers produced broad coagulation zones but also showed the highest degree of thermal spread, consistent with the combination of high NA and large mode volume.

The ablation dimensions obtained during the tests for the five flat-tip fibers are reported in Table 4.

The L and W values, together with the temperature increase, ΔT, between the initial and final temperatures, are reported as the mean ± standard deviation based on the number of samples tested for each experiment, as the initial temperature of the calf liver varied among the tests.

To ensure full comparability across all the experiments, all the tests were conducted on calf liver specimens belonging to the same batch and characterized by similar aging conditions. As a result of this experimental design, the number of measurements differed among the various fiber typologies, and in some cases only a single test was performed. For the configurations in which repeated measurements were carried out, the statistical analysis demonstrated highly consistent outcomes. In particular, the standard deviation associated with the geometrical characterization of the fibers remained within a 3–5% range across all the repeated tests, indicating that the selected biological substrate exhibits substantial uniformity and isotropy. This level of consistency supports the expectation that similar statistical behavior is also valid for the fiber types and test configurations for which only one measurement was available. To avoid introducing additional variability due to progressive tissue aging, the entire experimental campaign was therefore conducted using samples from the same batch. For those measurements in which a single sample was analyzed, the a priori error in the direct measurement of the two dimensions L and W was considered.

The test end time was determined by the selected power and the delivered dose, for example: Δt (3W@1800J) = 10 min, Δt (5W@1800J) = 6 min, and Δt (7W@1800J) = 4 min 17 s. The effective test duration was longer than the theoretical one due to an initial baseline temperature recording phase.

Across all the flat-tip fibers, the ablation size scaled with the delivered power (3 W→ 5 W → 7 W).

At higher energies (>1800 J), a saturation effect was noted: increases in L and W became progressively smaller, indicating heat diffusion-limited ablation growth.

**Figure 3:**
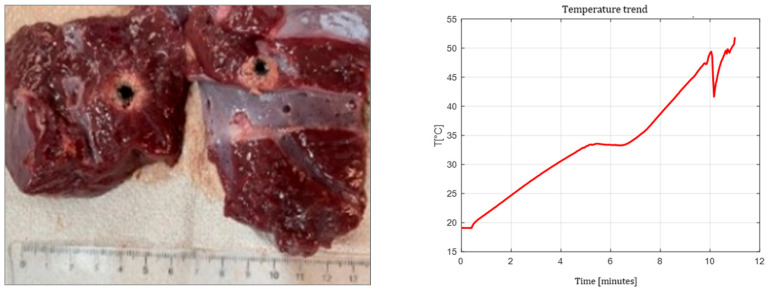
Single Fiber N°1. L = [18.0 ± 0.9] mm and W = [12.2 ± 1.0] mm (3W@1800J, 6 samples tested).

**Figure 4:**
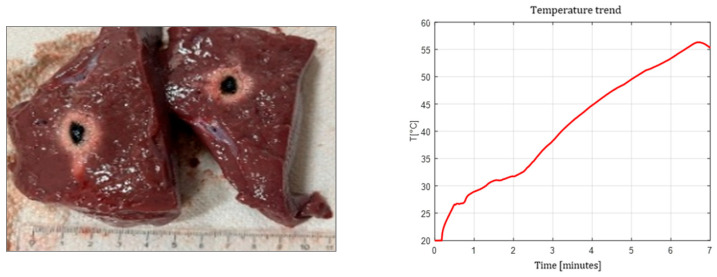
Single Fiber N°1. L = [19.3 ± 1.0] mm and W = [12.5 ± 0.6] mm (5W@1800J, 4 samples tested).

**Figure 5:**
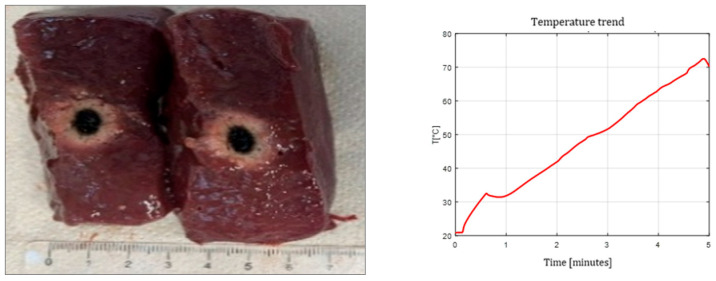
Single Fiber N°1. L = [23.0 ± 1.2] mm and W = [14.8 ± 1.0] mm (7W@1800J, 4 samples tested).

Figure 3, Figure 4 and Figure 5 show the effects of laser ablation on the calf liver and the temperature profiles recorded as a function of the test duration, using Fiber N°1. The resulting ablation dimensions were: L = [18.0 ± 0.9] mm and W = [12.2 ± 1.0] mm (3W@1800J, six samples tested); L = [19.3 ± 1.0] mm and W = [12.5 ± 0.6] mm (5W@1800J, four samples tested); L = [23.0 ± 1.2] mm and W = [14.8 ± 1.0] mm (7W@1800J, four samples tested).

**Figure 6:**
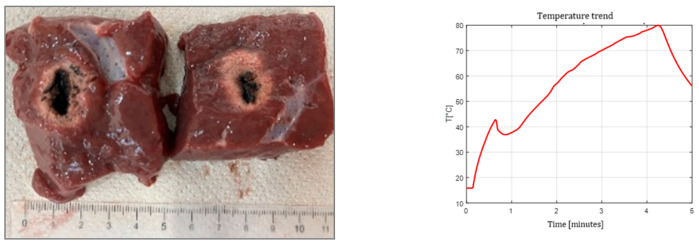
Single Fiber N°5 (7W@1800J, 2 samples tested). Ablation dimensions: L = [24.5 ± 0.7] mm and W = [16.0 ± 1.4] mm.

As an example, Figure 6 shows the ablation effects and the recorded temperature profile for Fiber N° 5 at 7W@1800J, which resulted in larger ablation dimensions compared to the other tests, in agreement with Table 4.

### 4.3. Radial Emission Conical Fibers

The fibers with conical diffusing tips (Fibers N°6–N°7, in Table 1) produced radially symmetric thermal profiles, with a significantly lower longitudinal extension compared to the flat-tip fibers.

1. The ablation shapes were more spherical and homogeneous.
2. The L decreased relative to the flat-tip fibers, confirming the lateral redistribution of energy.

**Figure 7:**
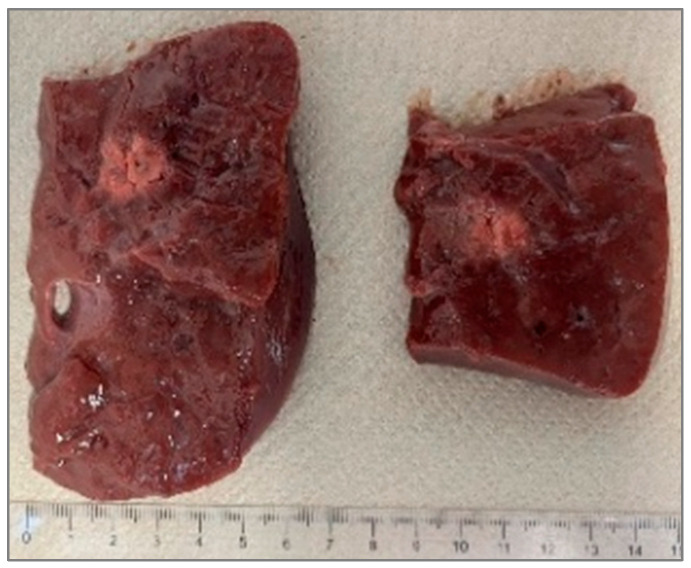
Single Fiber N°6 (3W@1800J, 1 sample tested). L = 13 mm and W = 13 mm.

The 400 µm radial fiber (Fiber N°7) produced slightly larger lesions than the 365 µm version (Fiber N°6), though the differences were less pronounced than in the flat-tip fibers, consistent with the dominating role of the diffuser. For these types of optical fibers, the temperature profile at a 10 mm distance from the tip was not analyzed, since the light radiation was not emitted in the axial direction, but laterally, according to the two radiation lobes. As can be seen in Figure 7, the ablation dimensions obtained, for example, in the 3W@1800J test with Fiber N°6 were L = 13.0 mm and W = 13.0 mm for a single sample tested.

### 4.4. Spherical Curved Ball-Tip Fiber

**Figure 8:**
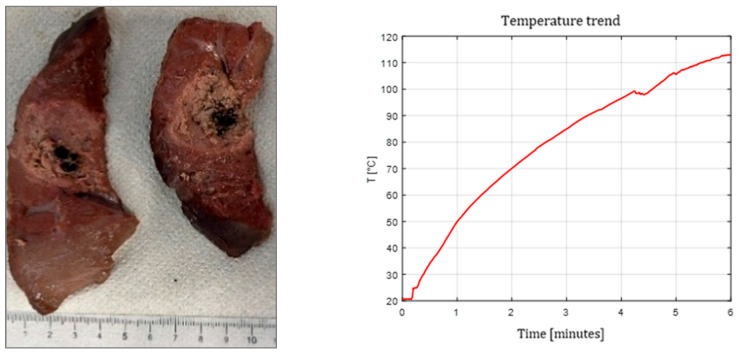
Single Fiber N°8 (5W@1800J, 1 sample tested). L = 27 mm and W = 20 mm.

The curved ball-tip fiber (Fiber N°8) generated highly isotropic emissions, producing ablations with the most uniform W/L ratio among all the fibers. The results show that, even with relatively low doses compared to the other tests (below 1800 J), setting the power to 5 W produces significantly larger ablation areas than those obtained with flat-tip fibers. As can be seen in Figure 8, the ablation dimensions obtained, for example, in the 5W@1800J test with Fiber N°8 were L = 27.0 mm and W = 20.0 mm for a single sample tested.

### 4.5. Temperature Profiles

The temperature measurements at 10 mm showed consistent trends:

1. The flat-tip fibers with a higher NA and higher core diameter reached peak temperatures faster.
2. The radial and ball-tip fibers exhibited lower initial temperature slopes, indicating slower local heating due to redistributed emissions.

Across all the fibers, increasing the laser power produced proportionally higher peak temperatures, though saturation effects were observed at 7 W.

### 4.6. Multi-Fiber and Pull-Back Configurations

Using two fibers at 10 mm spacing resulted in:

1. Substantial enlargement of the transverse ablation zone;
2. Partial merging of the two thermal fields;
3. Higher measured temperatures at the midpoint compared to the single-fiber trials.

**Figure 9:**
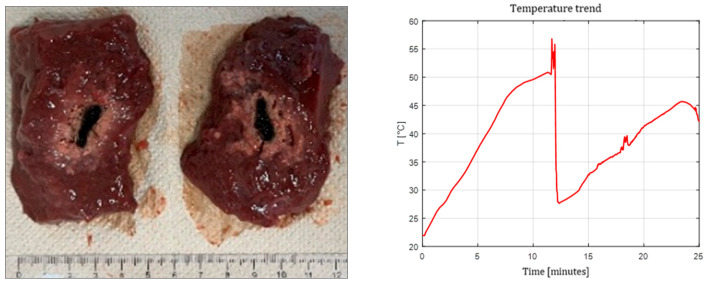
Single Fiber N°1 with pull-back. L = [27.0 ± 1.4] mm and W = 15 mm (3W@1800J, 2 samples tested).

**Figure 10:**
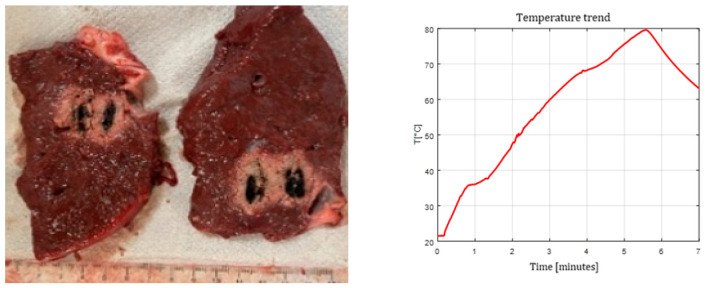
Double Fiber N°1 without pull-back. L = 20 mm and W = 25.5 mm (5W@1800J, 1 sample tested).

**Figure 11:**
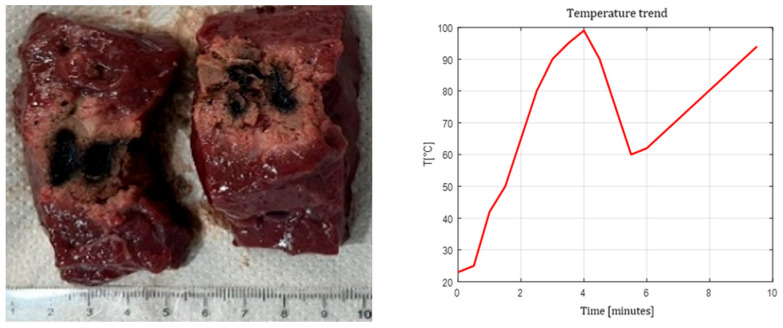
Double Fiber N°1 with pull-back. Ablation dimensions: L = 31 mm and W = 29 mm (7W@1800J, 1 sample tested).

The pull-back technique produced elongated lesions with cumulative lengths approximately equal to the sum of the two irradiation steps, providing a controllable method for extending ablation along the longitudinal axis. Figure 9, Figure 10 and Figure 11 show the effects of laser ablation on the calf liver and the temperature profiles recorded as a function of the test duration, using single and double Fiber N°1 with/without pull-back. The resulting ablation dimensions were: L = [27.0 ± 1.4] mm and W = 15 mm (3W@1800J, two samples tested, single Fiber N°1 with pull-back); L = 20 mm and W = 25.5 mm (5W@1800J, one sample tested, double Fiber N°1); L = 31 mm and W = 29 mm (7W@1800J, one sample tested, double Fiber N°1 with pull-back).

### 4.7. Balloon Catheter with Scattering Medium

**Figure 12:**
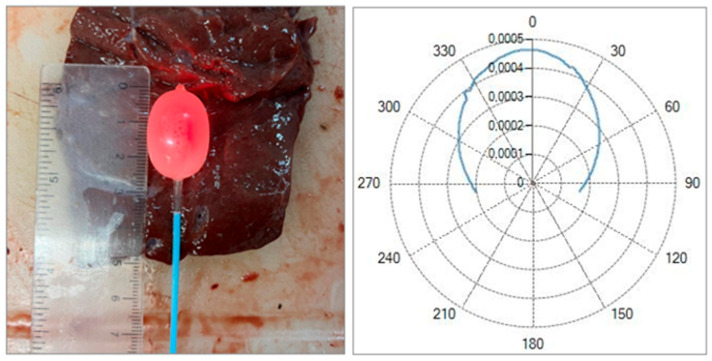
Balloon geometry of the catheter inflated with a solution containing 0.5 g of hydroxyapatite dispersed in 16 mL of saline solution at an internal pressure of 4 atm, and the corresponding radiation emission profile measured under the same conditions.

The preliminary tests with a balloon catheter filled with a hydroxyapatite suspension demonstrated the feasibility of generating more circular and homogeneous ablations. A photo of the catheter with the inflated balloon, alongside the calibration ruler, is shown in Figure 12. In this case, a red light was injected into the optical fiber to highlight the diffusive properties of the solution. It was observed that increasing the hydroxyapatite concentration improved the uniformity but reduced the overall transmitted power. The 0.2–0.5 g/16 mL mixture produced the best balance between uniformity and ablation extent.

These results suggest a viable approach for distributed circumferential ablation in confined anatomical spaces.

**Figure 13:**
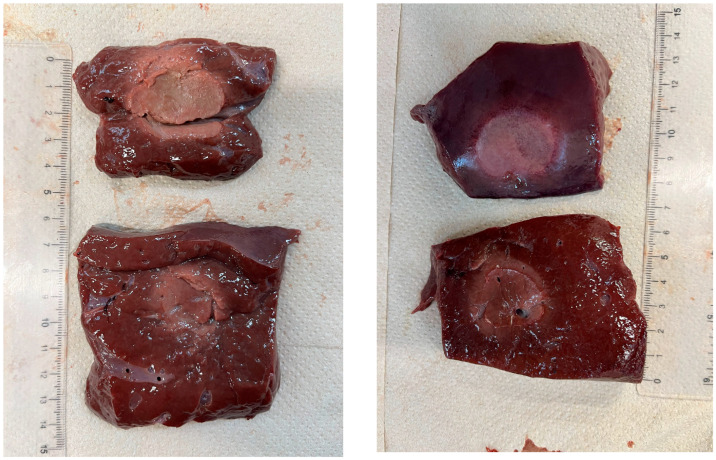
Balloon catheter test with Fiber N°1 at 7W@5400J. The balloon contains 0.2 g of hydroxyapatite dispersed in 16 mL of saline solution and is inflated to an internal pressure of 4 atm.

As can be seen in Figure 13, the ablation dimensions obtained for the 7W@5400J test with Fiber N°1 were L = [30.0 ± 1.4] mm and W = [25.5 ± 0.7] mm for two samples tested.

## 5. Discussion

This study provides a comparative analysis of eight multimode optical fibers for laser ablation with a 1064 nm Echolaser system, highlighting how the fiber geometry and optical properties directly influence the thermal distribution and lesion morphology.

### 5.1. Influence of Core Diameter and Numerical Aperture

The flat-tip fibers demonstrated that the ablation size grows with both the core diameter and *NA*, consistent with the broader emission cone and higher fluence delivered to the tissue.

The transition from NA 0.22 to 0.37 produced the largest relative gain in ablation width, confirming the NA as a dominant parameter in determining the lateral heat diffusion. These findings align with established models of interstitial laser therapy, where a higher NA increases the beam divergence and enlarges the radius of effective heating.

### 5.2. Radial and Spherical Emission for Uniform Ablation

Radial diffusers and the curved ball-tip fiber shifted the energy pattern from axial to isotropic emission, reducing longitudinal lesion elongation and producing more spherical coagulation volumes.

The radial fibers showed improved symmetry, supporting their potential use in anatomical regions where circumferential ablation is desired.

The ball-tip fiber produced the most extensive ablation dimensions compared to all the other fiber types.

### 5.3. Multi-Fiber and Pull-Back Strategies

The dual-fiber configuration highlighted how thermal fields can be combined to increase the ablation width, mirroring the clinical protocols used for prostate debulking. The pull-back method enabled longitudinal extension while preserving control over the local thermal deposition.

### 5.4. Temperature Evolution and Thermal Spread

The use of single-point temperature measurement was assumed to constitute a first investigation into the different type of fibers, and the dynamic temperature information was used only to estimate the thermal gradient at a fixed lateral-tip distance of 10 mm. We also assumed a homogeneous tissue without the presence of large vessels that could create asymmetry due to blood perfusion. The microthermocouple (Ø = 0.076 mm, length 2 m, max temp +260 °C) can achieve a resolution of 0.1 °C, accuracy in the order of ±0.3 °C using linearization, and is fast enough (time constant about 0.7 s) to follow the thermal gradients due to different thermal doses and fiber types.

The temperature measurements reflected the expected behavior of interstitial heating:

The large-core, high-NA fibers produced a faster temperature rise and higher steady-state values. The radial and spherical fibers heated more gradually, consistent with their diffused emission patterns.

The saturation effects regarding lesion growth and temperature at higher powers (7 W) suggest a regime where heat diffusion limits additional thermal spread.

The observed saturation plateau in the ablation volume arose from the physical mechanisms governing laser–tissue interaction and the intrinsic modality by which thermal ablation is produced. Laser energy is converted into heat only where optical absorption occurs, with the absorption coefficient playing the main role in the amount of laser intensity which is transformed into heat, inducing a local temperature increase. As tissue temperature exceeds ~60 °C, protein denaturation and coagulative necrosis develop, defining the ablation zone. At temperatures approaching 100 °C, water vaporization becomes a dominant energy sink: the high latent heat of vaporization and steam formation limit further temperature rise in adjacent tissue and reduce the efficiency of additional energy delivery for expanding the lesion [32]. Continued irradiation beyond tissue dehydration leads to carbonization, which markedly alters the tissue’s optical properties and drastically reduces light transmission, confining energy absorption to a thin superficial layer. At higher temperatures, sublimation of carbonized tissue generates a cavity around the laser applicator; as this cavity enlarges, the photon density decreases, and further sublimation becomes unsustainable. Under these conditions, additional laser energy is primarily dissipated by thermal conduction to the surrounding perfused tissue, which acts as an effective heat sink. Consequently, prolonged energy delivery no longer produces a proportional increase in the ablation volume, giving rise to the observed saturation plateau [33].

These findings can be interpreted with respect to a balance among laser energy deposition, thermal diffusion, and tissue carbonization. Flat-tip fibers deliver a higher forward-directed power density, which promotes deeper axial heating and results in larger lesions. However, lesion growth is not unlimited and becomes progressively constrained by thermal diffusion into the surrounding tissue and by surface carbonization, which can reduce the optical penetration and energy coupling. Increasing the core or NA slightly enlarges the illuminated area, moderating the local power density and thereby favoring energy transmission before carbonization occurs, resulting in larger lesions. In contrast, radial and spherical fiber tips distribute the laser power over a larger tissue volume, reducing the power density per unit area. This mitigates localized overheating and carbonization, while thermal diffusion dominates the heat spread, leading to more uniform ablation shapes.

### 5.5. Balloon Catheter Feasibility

The use of a balloon catheter with hydroxyapatite as a light-diffusing medium represents a novel approach not previously reported in the literature. The idea was to employ a biocompatible scatterer, commonly used in dentistry, to diffuse the laser radiation within the balloon and achieve more uniform and circular ablations. This approach aims to enable selective tissue removal within a body cavity.

The radiation pattern reported in the manuscript demonstrates the effect of the hydroxyapatite diffuser. A flat-tip optical fiber (Fiber N°1), which normally produces a single lobe of radiation, was placed inside the balloon. In the presence of the hydroxyapatite powder, the radiation lobe became wider and more isotropic, showing that the diffuser increased the spread of laser energy.

During the tests, it was observed that the hydroxyapatite powder tended to settle on one side of the balloon due to gravity and the micrometric size of the particles. As a result, some regions of tissue received more thermal damage, while the symmetrically opposite areas with powder accumulation experienced reduced heating due to increased attenuation.

Despite these limitations, this balloon catheter configuration represents a promising concept for future developments. Its performance could be improved by using finer hydroxyapatite powders to reduce sedimentation and further enhance uniform tissue ablation.

The balloon catheter study provides preliminary evidence that combining a scattering medium with a flat-tip fiber can enable controlled, symmetric ablations in constrained environments. Although the transmitted power decreases with the concentration, the improved uniformity may benefit applications requiring circumferential or cylindrical ablations. This concept could be relevant for endocavitary laser procedures or minimally invasive treatments where preserving the tissue symmetry is essential.

### 5.6. Limitations

The experiments were performed ex vivo, without perfusion, likely overestimating the thermal lesion size compared to in vivo conditions. Tissue variability and dehydration may have introduced minor inconsistencies among the samples.

In vivo, perfusion acts as a dynamic heat sink, influencing the temperature distribution and ablation extent, particularly at the lesion periphery; therefore, from a safety perspective, the ablation volumes obtained ex vivo are expected to overestimate those achievable in perfused tissue under equivalent energy delivery. Nevertheless, ex vivo experiments remain valuable for comparative evaluations of fiber designs, as they isolate the intrinsic thermo-optical interaction between laser emission and tissue. A quantitative translation to in vivo conditions requires accounting for perfusion-mediated heat dissipation, which can be addressed through perfusion-inclusive bioheat transfer models, such as the Pennes equation [34]. Previous studies have shown comparable results between ex vivo ablation at 20 °C and in vivo ablation at 37 °C with perfusion [35], suggesting that the lower initial tissue temperature in ex vivo models may partially compensate for the absence of blood perfusion. Moreover, ex vivo ablation results have been shown to be consistent with theoretical models combining the Pennes bioheat equation, Beer–Lambert law for optical absorption, and Arrhenius damage modelling [36,37], further supporting the validity of the experimental approach.

## 6. Conclusions

This study provides a systematic experimental assessment of eight optical fibers with different core diameters, numerical apertures, and tip geometries for soft-tissue ablation using a 1064 nm Echolaser system. The fiber characteristics were shown to strongly influence the ablation dimensions, temperature evolution, and lesion uniformity. Flat-tip fibers with large core diameters and a high NA produced the greatest ablation volumes, while radial and spherical diffusers generated more isotropic and homogeneous lesions.

Multi-fiber and pull-back techniques enabled the controlled enlargement of the coagulation zone, and the preliminary balloon catheter tests demonstrated a promising approach for uniform circumferential ablations. These findings provide a quantitative reference framework for selecting and designing optical fibers tailored to specific minimally invasive urological applications, while supporting the future optimization of laser-based therapeutic devices.

Future research should focus on integrating real-time temperature mapping or thermographic imaging to enhance intraoperative monitoring, optimizing balloon catheter designs for in vivo applications, and evaluating fiber durability under repeated clinical use. Such developments could further improve the safety and reproducibility of fiber-based laser ablation therapies.

## Figures and Tables

**Table 1:** Characteristics of tested optical fibers.

Fiber ID	Core [µm]	OD [µm]	NA	Tip Geometry	Manufacturer
N°1	272	420	0.22	Flat tip	Oberon–Elesta custom
N°2	300	350	0.22	Flat tip	Asclepion
N°3	300	650	0.37	Flat tip	Thorlabs
N°4	400	730	0.37	Flat tip	Oberon
N°5	600	1040	0.48	Flat tip	Thorlabs
N°6	365	800	0.22	Conical tip	Oberon–Elesta custom
N°7	400	950	0.22	Conical tip	Oberon
N°8	600	890	0.22	Curved ball tip	Oberon

**Table 2:** Radiation profiles of the eight types of optical fibers analyzed.

Fiber ID	Radiation Profiles
N°1–N°5	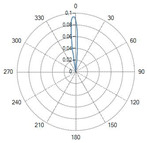 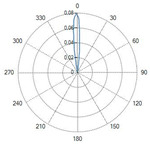 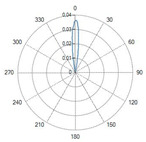 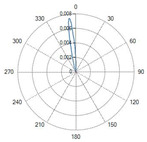 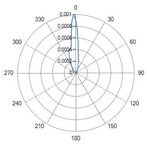
N°6 and N°7	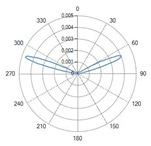 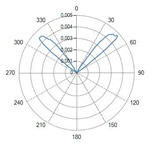
N°8	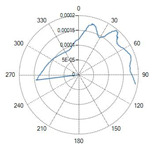

**Table 3:** Thermal and optical properties of tissues: prostate, calf liver and porcine muscle.

	Thermal Properties			% Water	Optical Properties (*λ* = 1064 nm)	
	Tissue Density [kg·m^−3^]	Specific Heat [J·kg^−1^·°C^−1^]	Thermal Conductivity [W·m^−1^·°C^−1^]		Absorption Coefficient [cm^−1^]	Diffusion Coefficient [cm^−1^]
Prostate	1045	3715	0.51	82	0.4	110
Calf liver	1079	3540	0.52	75–81.9	0.3–0.5	150–169
Porcine muscle	1082–1100	3490	0.44–0.49	73–75	0.12–0.22	2.5–5.0

**Table 4:** Ablation dimensions (L, W) and ΔT in single flat tip fiber tests, Ns number of samples.

Fiber ID	3W@1800J	5W@1800J	7W@1800J
N°1	L = [18.0 ± 0.9] mm W = [12.2 ± 1.0] mm ΔT = [30.9 ± 4.3] °C Ns = 6	L = [19.3 ± 1.0] mm W = [12.5 ± 0.6] mm ΔT = [34.1 ± 3.2] °C Ns = 4	L = [23.0 ± 1.2] mm W = [14.8 ± 1.0] mm ΔT = [48.0 ± 9.8] °C Ns = 4
N°2	L = [20.0 ± 0.5] mm W = [13.0 ± 0.5] mm ΔT = [40.1 ± 0.3] °C Ns = 1	L = [22.0 ± 0.5] mm W = [14.0 ± 0.5] mm ΔT = [45.4 ± 0.3] °C Ns = 1	L = [23.5 ± 0.7] mm W = [14.0 ± 0.5] mm ΔT = [66.4 ± 0.3] °C Ns = 2
N°3	L = [20.5 ± 0.7] mm W = [15.5 ± 0.7] mm ΔT = [34.2 ± 4.7] °C Ns = 2	L = [22.5 ± 0.7] mm W = [15.5 ± 0.7] mm ΔT = [41.6 ± 0.3] °C Ns = 2	L = [24.5 ± 0.7] mm W = [17.0 ± 1.4] mm ΔT = [56.6 ± 0.3] °C Ns = 2
N°4	L = [20.0 ± 0.5] mm W = [13.5 ± 0.7] mm ΔT = [38.3 ± 5.6] °C Ns = 2	L = [23.0 ± 0.5] mm W = [15.0 ± 0.5] mm ΔT = [40.7 ± 4.5] °C Ns = 2	L = [28.0 ± 0.5] mm W = [17.0 ± 0.5] mm ΔT = [54.7 ± 0.3] °C Ns = 1
N°5	L = [21.5 ± 0.7] mm W = [15.5 ± 0.7] mm ΔT = [45.3 ± 1.6] °C Ns = 2	L = [23.0 ± 1.0] mm W = [16.0 ± 1.0] mm ΔT = [55.3 ± 0.3] °C Ns = 2	L = [24.5 ± 0.7] mm W = [16.0 ± 1.4] mm ΔT = [64.2 ± 0.3] °C Ns = 2

## Data Availability

The data used to support the findings of this study are available from the corresponding author upon request.
